# Alterations of Gab2 signalling complexes in imatinib and dasatinib treated chronic myeloid leukaemia cells

**DOI:** 10.1186/1478-811X-11-30

**Published:** 2013-04-22

**Authors:** Sebastian Halbach, Kristoffer TG Rigbolt, Franziska U Wöhrle, Britta Diedrich, Christine Gretzmeier, Tilman Brummer, Jörn Dengjel

**Affiliations:** 1 Institute of Molecular Medicine and Cell Research (IMMZ), Faculty of Medicine, Albert-Ludwigs-University Freiburg, Stefan-Meier-Str. 17, Freiburg 79104, Germany; 2 Centre for Biological Systems Analysis (ZBSA), Albert-Ludwigs-University Freiburg, Habsburgerstraße 49, Freiburg 79104, Germany; 3 Institute for Biology III, Faculty of Biology, Albert-Ludwigs-University Freiburg, Schänzlestraße 1, Freiburg 79104, Germany; 4 Spemann Graduate School of Biology and Medicine, Albertstraße 19A, Freiburg 79104, Germany; 5 Centre for Biological Signalling Studies BIOSS, Schänzlestraße 18, Freiburg 79104, Germany; 6 Freiburg Institute for Advanced Studies (FRIAS), Albert-Ludwigs-University Freiburg, Albertstraße 19, Freiburg 79104, Germany; 7 Comprehensive Cancer Center Freiburg (CCCF), Universitätsklinikum Freiburg, Hugstetter Straße 55, Freiburg 79106, Germany

**Keywords:** Chronic myeloid leukaemia, Bcr-Abl, Gab2, Tyrosine kinase inhibitor, Imatinib, Dasatinib, SILAC-based mass spectrometry, Proteomics, Casein kinase, Protein phosphorylation

## Abstract

**Background:**

The Gab2 docking protein acts as an important signal amplifier downstream of various growth factor receptors and Bcr-Abl, the driver of chronic myeloid leukaemia (CML). Despite the success of Bcr-Abl tyrosine kinase inhibitors (TKI) in the therapy of CML, TKI-resistance remains an unsolved problem in the clinic. We have recently shown that Gab2 signalling counteracts the efficacy of four distinct Bcr-Abl inhibitors. In the course of that project, we noticed that two clinically relevant drugs, imatinib and dasatinib, provoke distinct alterations in the electrophoretic mobility of Gab2, its signalling output and protein interactions. As the signalling potential of the docking protein is highly modulated by its phosphorylation status, we set out to obtain more insights into the impact of TKIs on Gab2 phosphorylation.

**Findings:**

Using stable isotope labelling by amino acids in cell culture (SILAC)-based quantitative mass spectrometry (MS), we show now that imatinib and dasatinib provoke distinct effects on the phosphorylation status and interactome of Gab2. This study identifies several new phosphorylation sites on Gab2 and confirms many sites previously known from other experimental systems. At equimolar concentrations, dasatinib is more effective in preventing Gab2 tyrosine and serine/threonine phosphorylation than imatinib. It also affects the phosphorylation status of more residues than imatinib. In addition, we also identify novel components of the Gab2 signalling complex, such as casein kinases, stathmins and PIP1 as well as known interaction partners whose association with Gab2 is disrupted by imatinib and/or dasatinib.

**Conclusions:**

By using MS-based proteomics, we have identified new and confirmed known phosphorylation sites and interaction partners of Gab2, which may play an important role in the regulation of this docking protein. Given the growing importance of Gab2 in several tumour entities we expect that our results will help to understand the complex regulation of Gab2 and how this docking protein can contribute to malignancy.

## Lay abstract

Chronic myeloid leukaemia (CML) is characterized by the increased and deregulated growth of myeloid cells in the bone marrow and their accumulation in the peripheral blood. CML represents about 20% of all cases of adult leukaemia and has an incidence rate of 1.6 per 100,000 people per year. This form of leukaemia is staged into three distinct phases. The first phase (chronic phase, CP) is often asymptomatic and progresses over several years to the accelerated phase (AP), if undiscovered or left untreated. The AP ultimately progresses to the terminal blast crisis (BC) which is characterized clinically like an acute leukaemia. CML is driven by the oncogenic Bcr-Abl tyrosine kinase. This kinase originates from the spontaneous rearrangement of two human chromosomes (Philadelphia chromosome) causing the production of a chimeric protein with combined biological portfolios. The Bcr-moiety recruits novel interaction partners, which are modified on tyrosine residues with phosphate groups by the Abl moiety, a typical protein tyrosine kinase. This in turn activates numerous intracellular signalling pathways that are, under physiological circumstances, only activated by growth signals. As a result, cells, which harbour the Philadelphia chromosome, grow excessively, even in the absence of growth signals and thereby cause CML. This insight led to the development of drugs inhibiting the Bcr-Abl enzyme, which have revolutionised CML therapy. In some patients, however, these drugs do not cause remission or initially responsive leukaemia become drug resistant. Among several mechanisms, dysregulation of signal transducers downstream of Bcr-Abl, such as the Gab2 docking protein, contribute to drug resistance. In this manuscript, we provide a detailed analysis on the phosphorylation status and protein interaction repertoire of Gab2 in CML cells in the presence and absence of the clinically applied drugs imatinib (Gleevec) and dasatinib (Sprycel).

## Background

The oncogenic protein tyrosine kinase Bcr-Abl, the product of the Philadelphia chromosome, drives chronic myeloid leukaemia (CML) [1]. This disease can be classified into three distinct stages consisting of an often asymptomatic chronic phase (CP), with less than 10% blasts in the bone marrow, followed by an accelerated phase (AP) and finally blast crisis (BC), with more than 20% blasts [2]. The latter phase resembles an acute leukaemia and is usually fatal, if left untreated. The discovery of Bcr-Abl and the subsequent elucidation of its structure and mode of action led to the development of the tyrosine kinase inhibitor (TKI) imatinib mesylate (IM) [3]. IM is an ATP-competitive inhibitor and owes its success to the exploitation of oncogene addiction, a phenomenon in which a tumour cell has become critically dependent on a single oncogenic pathway for its proliferation and/or survival [4-6]. Consequently, inhibition of Bcr-Abl signalling provokes severe “withdrawal” symptoms and tumour regression.

Despite the useful application of IM in cancer therapy, only 3 out of 4 CML cases respond adequately to IM in the first place and several of the initially responding tumours become TKI resistant due to the acquisition of Bcr-Abl mutations preventing TKI uptake and/or action [4]. This insight spurred the development of additional Bcr-Abl inhibitors such as the ATP-competitive compound dasatinib (DST). Bcr-Abl mutation-independent mechanisms of TKI-resistance are also of clinical importance, but remain ill-defined at the molecular level. However, there is increasing evidence that the aberrant activity or expression of components of the Bcr-Abl signalling network contribute to TKI-resistance [7,8]. Consequently, the combination of Bcr-Abl inhibitors with inhibitors of other signalling pathways may improve the treatment of therapy-resistant CML [8,9]. Nevertheless, such strategies will require an in-depth knowledge of the individual components and their contribution to the Bcr-Abl signalling network.

The fusion of the *BCR* and *ABL* reading frames extends the portfolio of the Abl kinase by interaction partners of the Bcr moiety such as the Grb2 adaptor [1,10]. As a consequence, Bcr-Abl organises a multimeric protein complex and activates various signalling pathways [11,12]. One critical signal transducer of Bcr-Abl and Grb2 interaction partner is the docking protein and proto-oncogene product Gab2 [13,14]. Grb2 is connected *via* its central SH2 domain to phospho-tyrosine 177 (Y177) in the Bcr moiety, while its C-terminal SH3 domain binds to a typical and an atypical Grb2 binding site in Gab2 [10,15,16]. This “Grb2 bridge” is essential for the transformation of murine myeloid progenitors and for the prominent tyrosine phosphorylation of Gab2 in Bcr-Abl transformed cells [9,17]. These phospho-tyrosine residues act as docking sites for various effectors with SH2 domains such as the tyrosine phosphatase Shp2 and the regulatory p85 subunit of PI3K [13]. The critical function of these residues was demonstrated by the use of signalling-impaired Gab2 mutants in which the phosphorylation of these docking sites was prevented by blocking the Grb2/Gab2 interaction or by replacing the critical tyrosines by non-phosphorylatable phenylalanine residues [9,17-20]. Upon Gab2 tyrosine phosphorylation downstream effectors then mediate the amplification of Bcr-Abl derived signals through the Ras/ERK and PI3K/AKT/mTOR pathways. The activation of these pathways can lead to uncontrolled proliferation and survival in this and other settings, in which aberrant Gab2 signalling contributes to tumourigenesis [9,13,14]. In addition to the relatively well-characterised tyrosine phosphorylation sites, Gab2 is phosphorylated on more than 20 Ser/Thr-residues, whose regulatory function remains mostly unknown [19]. However, four sites (S159, S210, T391 and S623) fulfil important roles in downregulating Gab2 signalling output by three distinct negative feedback loops [19,21-23].

The important role of Gab2 downstream of Bcr-Abl is illustrated by the observations that its genetic depletion prevents the transformation of murine myeloid progenitors by the fusion kinase [17] and slows down the proliferation of primary human CML cells and the CML cell line K562 [9,24]. Furthermore, there is increasing evidence that Gab2 expression levels or the abundance of cells with prominent expression of the docking protein increase during CML progression from chronic phase to blast crisis [25,26]. Importantly, we have recently shown in various CML model systems that Gab2 signalling confers resistance to multiple Bcr-Abl selective TKIs [9]. In this study, we demonstrated that IM and DST provoke distinct alterations of the electrophoretic mobility and signalling output of Gab2 suggesting that these drugs impact differently on post-translational modifications, like the phosphorylation status, of the docking protein. These findings prompted us to conduct a more detailed characterisation of the Gab2 signalling complex in response to the TKIs IM and DST by quantitative mass spectrometry (MS).

Stable isotope labelling by amino acids in cell culture (SILAC) is a metabolic labelling strategy allowing, amongst other things the quantitative investigation of posttranslational modification (PTM) [27], protein-protein interactions [28], protein turnover [29] and organellar compositions changes [30] by unbiased MS-based proteomics approaches. Using SILAC-based MS, we show now on which sites and to which extent both drugs affect the phosphorylation status of Gab2. Additionally, we document the impact of these drugs on the composition of Gab2-mediated signalling complexes.

## Results and discussion

In order to obtain more insights into the plasticity of phosphorylation and protein interaction events in the Gab2 signalling complex in the context of Bcr-Abl signalling, we made use of our previously established model systems, the K562tet/Gab2-HA cell line and the murine pro-B cell line Ba/F3 expressing p210^Bcr-Abl^ ectopically [9]. K562tet/Gab2-HA cells are derived from the K562 cell line, which was established from a CML patient in blast crisis and is widely used for the development of Bcr-Abl inhibitors [31]. K562tet/Gab2-HA cells are fitted with an improved doxycycline (dox)-inducible system for the expression of haemagglutinin (HA)-tagged human Gab2 [9,32], which can be efficiently purified for proteomic analyses [19]. Although overexpression of Gab2-HA may influence protein interactions, we only compare data generated in the same isogenic cellular system. Hence, expression differences should not affect our findings.

**Figure 1:**
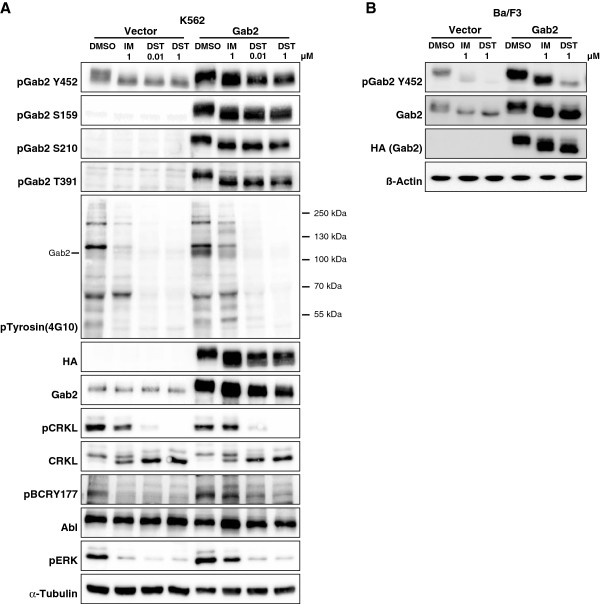
**Inhibitor-dependent Gab2 phosphorylation.** (**A**) K562tet cells stably transfected with the inducible Gab2^wt^ expression vector or ‘empty’ control vector [9] were treated with 1 μg/ml dox for 24 h and then exposed to 1 μM imatinib or 0.01 μM and 1 μM dasatinib or to the same volume of DMSO (vehicle control) for 4 h. Total cellular lysates were analysed by western blotting using the indicated antibodies. (**B**) The murine proB cell line Ba/F3 was transduced with pBabe-Hygro-Bcr-Abl and subsequently with either pWZL-Bsr-Gab2-HA, or pWZL-Bsr empty control vector [9]. Cells were exposed to either 1 μM imatinib or dasatinib or to the same volume of DMSO (vehicle control) for 1 h. Total cellular lysates were analyzed by western blotting using the indicated antibodies.

To gain more insights into the phosphorylation status of Gab2 in Bcr-Abl positive cells, we first used phospho-specific antibodies raised against phospho-tyrosine in general (pY), pY452 (PI3K recruitment) and the PI3K dependent feedback phosphorylation sites S159, S210 and T391 [19,21]. This analysis (Figure 1) showed that applications of the two clinically relevant CML drugs, IM (1 μM) and DST (0.01 and 1 μM), provoke distinct alterations in the electrophoretic mobility of Gab2, a rough measure of its phosphorylation status. The concentration of 1 μM IM was chosen as it is in the range of the observed trough plasma levels of CML patients receiving standard doses of IM (1.76-2.95 μM; [33]) and is widely used to study IM induced cell death in K562 cells [9,34,35]. Although patients in chronic phase contain DST plasma levels 30-fold lower than the drug concentrations chosen for our experiments, we decided to use also 1 μM DST as we have previously shown that not all Gab2 amplified signalling events, e.g. AKT phosphorylation, were abolished by this concentration, and importantly, exogenous Gab2 was still able to protect K562 cells against 1 μM DST [9]. However, to account for the different IC_50_ values of the two drugs, we also included a second DST concentration, 0.01 μM, which lies in the range of observed plasma levels of DST, as well. The accelerated electrophoretic mobility of Gab2 was accompanied by a drastic reduction in its overall tyrosine phosphorylation, while the specific phosphorylation of Y452 was quite resilient in K562 cells (Figure 1A) as reported previously [9]. Interestingly, 0.01 μM DST, which exhibited a similar potency in Bcr-Abl inhibition as 1 μM IM according to previous findings [36] and our own data (compare the degree of Bcr-Abl autophosphorylation at Y177 in Figure 1A between lanes 1 and 4), yielded a distinct pattern of overall tyrosine phosphorylation than IM and was more potent in reducing CrkL and ERK phosphorylation (Figure 1A). Thus, despite equi-potent inhibition of Bcr-Abl, IM and DST provoke distinct alterations of phosphorylation events, which can be explained by the broader target spectrum of the latter [37].

In agreement with our previous finding that both IM and DST hardly block AKT phosphorylation in K562 cells [9], we show here for the first time that S159 phosphorylation is not abrogated by either the drugs. Likewise, S210 and T391, which serve as binding sites for 14-3-3 proteins and whose phosphorylation is stimulated by PI3K [19], display more or less constitutive phosphorylation (Figure 1A). While our work was in progress, Preisinger et al. (2013) also reported that Gab2 is bound to various 14-3-3 members under basal conditions and that even a dose of 10 μM IM does not disrupt these interactions [38]. Furthermore, a certain level of inhibitory Gab2/14-3-3 complexes under steady state conditions can be inferred not only from the data in Figure 1 but also from our previous report showing that the Gab2^S210A/T391A^ double mutant (Gab2^2xA^), which cannot recruit 14-3-3 proteins [19], displays an enhanced signalling potential in K562 cells [9]. Taken together, even high doses of DST have minimal impact on the three PI3K dependent phosphorylation sites on Gab2 and its 14-3-3 binding potential. This is of particular interest as a recent study, primarily conducted in HEK293 cells, provided convincing evidence that the ERK/RSK axis can drive the phosphorylation of S159 and S210 as well [39]. However, the fact that we still observe substantial amounts of S159 and S210 phosphorylation despite the drastic loss of ERK phosphorylation suggests that the contribution of the ERK pathway plays a minor role in phosphorylation of these sites in K562 cells.

**Figure 2:**
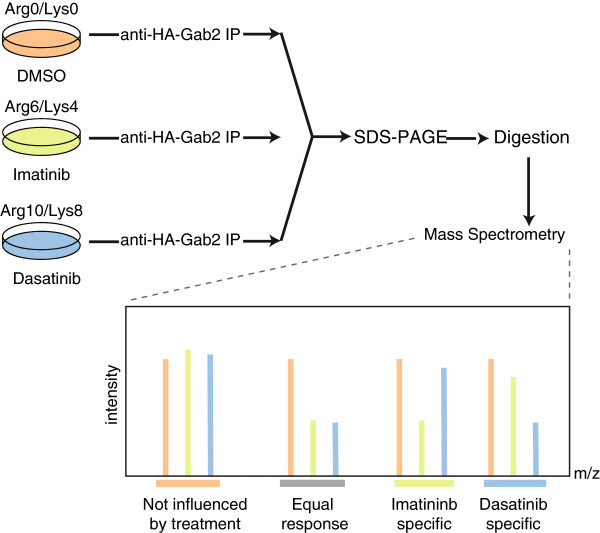
**Quantitative proteomics workflow.** Differentially SILAC labeled K562 cells were treated for 4 h with either 1 μM imatinib or 0.01 μM and 1 μM dasatinib, and DMSO as control, respectively. Cells were lysed, Gab2 protein complexes purified by anti-HA sepharose, complexes eluted and combined 1:1:1 for further analysis. Proteins were separated by SDS-PAGE, in-gel digested using trypsin, and resulting peptide mixtures analyzed by LC-MS/MS. A biological replicate with reversed labels was performed. Specific Gab2 interactors and proteins binding unspecific to beads and antibody will be present in a 1:1:1 ratio. Protein interactions dependent on inhibitor sensitive phosphorylation sites will be reduced.

**Figure 3:**
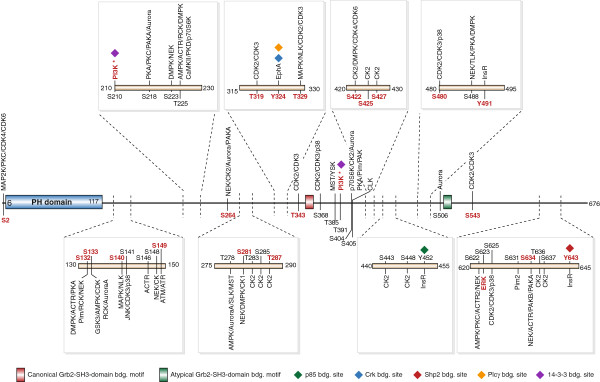
**The Gab2 phosphomap in K562 cells.** Shown is a representation of the Gab2 structure indicating domains, protein binding motifs and sites. Phosphosites identified in this study are annotated in black, sites that respond more than two fold to imatinib and/or dasatinib in red. Kinases known to phosphorylate a specific site are annotated in red, predicted kinases using NetworKIN [53] in black. * means PI3K dependent.

Moreover, the drastic increase in the electrophoretic mobility of Gab2 in DST treated cells implies that many more phosphorylation events are affected. Indeed, we had shown previously that Gab2 is phosphorylated on more than 20 Ser/Thr-residues in EGF-stimulated mammary epithelial cells [19]. To conduct a quantitative direct comparison of Gab2 signalling complexes purified from IM or DST treated cells, the K562tet/Gab2-HA line was subject to a SILAC labelling protocol (Figure 2). The cells were cultured with light (Arg0/Lys0), medium (Arg6/Lys4) and heavy (Arg10/Lys8) labelled amino acids for ten days and Gab2 overexpression was induced 24 h prior to the treatment with 1 μM IM or DST (see Methods for details). To exclude labelling artefacts, a biological replicate with swapped labels was performed. In addition, a second set of experiments was performed comparing 0.01 μM to 1 μM DST to account for the different potencies of IM and DST. Gab2 complexes were purified by immunoprecipitation, pooled and analysed by MS. In addition to the previously identified residues, we identified 18 novel phosphorylation sites (Figure 3) raising the tally to 72 *bona fide* phosphorylation sites (Table 1). Interestingly, apart from Y48/Y49, which were not identified in our studies, none of the known phosphorylation sites maps to the PH domain, but only to the very N-terminus and the long disordered C-terminal tail (Figure 3). This observation fits well to the notion that phosphorylation sites usually occur within intrinsically disordered protein regions [40,41]. It should be also noted that, in contrast to the recent study by Preisinger et al. [38], we did not perform an enrichment for phospho-tyrosine containing peptides as this procedure could discriminate against Ser/Thr-phosphorylated residues, which often represent the outcome of negative feedback loops in tyrosine kinase signalling [19,21-23,39]. The wealth of Gab2 phosphorylation sites raises the question which kinases and phosphatases control these post-translational modifications and identifies areas for future research. As indicated in Figure 3 and in Additional file 1: Table S1, software algorithms such as NetworKin [42] implicate several kinases in these processes including CK2. In order to provide a more comprehensive Gab2 phospho-map, we combined our data with those of previous publications and the curated Phosphosite database (Table 1). Interestingly, this “meta-map” of Gab2 phosphorylation sites (Table 1) reveals a largely isoform-specific cluster of phosphorylated Ser/Thr-residues (S622-S637) preceding the Shp2 recruitment motif around Y643. It is tempting to speculate that these phosphorylation events affect the Gab2/Shp2 interaction as it was already discussed for the single residues S623 [22] and S631 [39].

**Table 1:** A meta-map of Gab2 phosphorylation

**Residue**	**This study**	**IM-treated K562 cells [**[38]**]**	**EGF-stimulated MCF-10A cells [**[19]**]**	**Phospho-sitePlus**	**Protein docking site or other known function**	**Conserved in Gab1**
**S2**	X					
Y48		X		X		X
Y49				X		X
**S132**	X					
S133	X		X	X		
S140	X		X	X		
S141	X		X	X		
**S146**	X					
S148	X		X	X		
S149	X		X	X		
S159	Western (Figure 1A)		X	X	Negative regulatory site; AKT substrate [21]	
S164			X	X		
T171				X		X
Y194				X		
S210	X		X	X	14-3-3 binding and negative regulatory site [19,23]	
S218	X		X	X		X
S223	X		X	X		
**T225**	X					
Y249				X	CrkL/PLCγ [13]	X
S250				X		X
S264	X		X	X		X and phosphorylated [57]
T265				X		X
Y266		X		X	CrkL/PLCγ [13]	X
T278	X		X			
S281	X		X	X		
**T283**	X					
**S285**	X					
T287	X		X	X		
Y293		X	X	X	CrkL [13]	X
T294				X		
**T319**	X					X
**Y324**	X				CrkL/PLCγ [13]	X
**T329**	X					X
T331			X			X
**T343**	X					
**S368**	X					X
T385	X		X	X		
T391	X		X	X	14-3-3 binding and negative regulatory site [19,23]	X and phosphorylated [57]
S404	X			X		X
S405	X		X	X		X
T408				X		
Y409		X		X		X
Y411				X		
**S422**	X					X and phosphorylated [57]
**S425**	X					
**S427**	X					
**S443**	X					
**S448**	X					
Y452	X			X	p85 recruitment site; Resilient against IM and DST in K562 cells [9]	X
S472					X	X
S474					X	X
Y476					X	X
S480	X		X	X		X
S488	X			X		X
Y491	X			X		
**S506**	X					
S543	X		X	X	ERK dependent phosphorylation of the S543 equivalent critical for PH-domain mediated membrane recruitment of Gab1 [56]. ERK consensus motif (PxxSP) conserved in Gab2	X
S550				X		
Y563				X		
Y584				X	p85 [13]	X
S611				X		
Y614				X	Shp2 [13]	X
S622	X		X	X		X
S623	X		X	X		
S625	X			X		X
S631	X			X		
T632				X		
S633				X		
**S634**	X					
T636	X			X		X
S637	X			X		
Y643	X			X	Shp2 [13]	X

Comparison of the phospho-maps of Gab2 from this study with those established for IM treated K562 cells [38], EGF stimulated MCF-10A cells [19] and the sites listed in the curated phosphosite database (http://www.phosphosite.org). Note that in addition to other entries, the entries from the MCF-10A cells are also represented in this database. See Wöhrle et al. (2009) for further details on the indicated docking sites. Gab2 phoshorylation sites that have a homotopic counterpart in Gab1 (incl. conserved S⇔T conversions) were identified by comparing the human Gab2 (NP_53679) and Gab1 (NP_997006.1) sequences using the BLASTP 2.2.27+ tool. However, it should be noted that in many cases only the phosphorylation site itself and not its context were conserved between both paralogues, like it was described for the T391/T387 in Gab2/Gab1 [19,57]. All new Gab2 phosphorylation sites, identified in this study were marked in bold.

**Figure 4:**
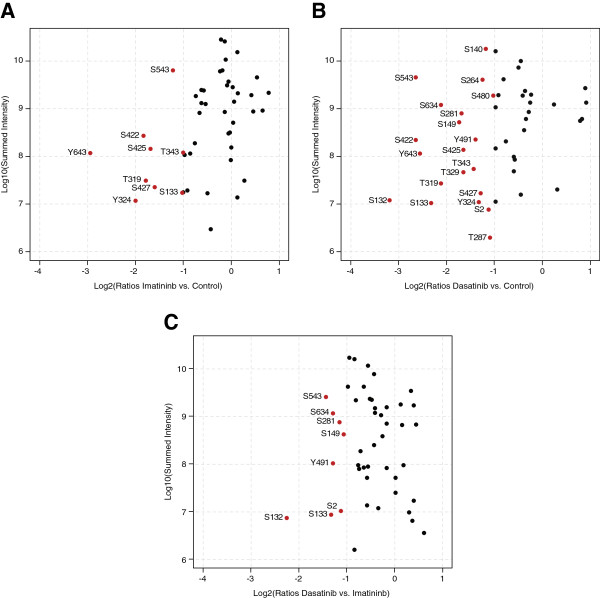
**The phosphorylation status of Gab2 is differentially affected by imatinib and dasatinib. (A-B)** Plotted are Gab2 phosphosite ratios normalized to Gab2 protein ratios of imatinib, dasatinib versus control treated cells of two biological replicates against respective summed peptide intensities. Sites changing more than two fold are annotated and marked in red. (**C**) Same set-up as for (**A**-**B**) except that Gab2 phosphosites from imatinib treated cells are compared with those from dasatinib treated cells.

**Figure 5:**
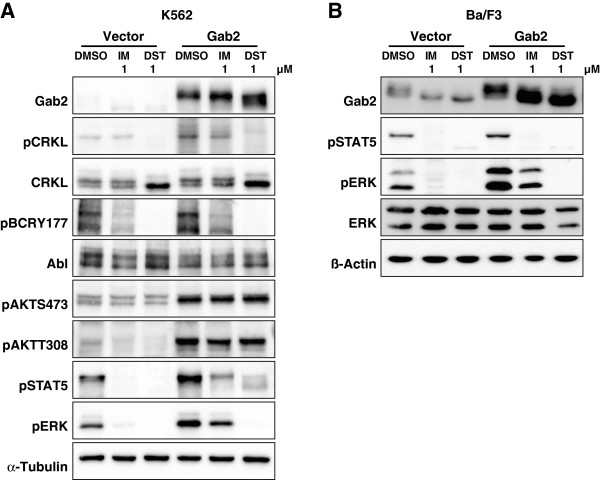
**Inhibitor-dependent effects on Bcr-Abl and Gab2 downstream effectors.** (**A**) K562tet cells stably transfected with the inducible Gab2^wt^ expression vector or ‘empty’ control vector [9] were treated with 1 μg/ml dox for 24 h and then exposed to either 1 μM imatinib or dasatinib or to the same volume of DMSO (vehicle control) for 4 h. Total cellular lysates were analyzed by Western blotting using the indicated antibodies. (**B**) The murine proB cell line Ba/F3 was transduced with pBabe-Hygro-Bcr-Abl and subsequently with either pWZL-Bsr-Gab2-HA, or pWZL-Bsr empty control vector [9]. Cells were exposed to either 1 μM imatinib or dasatinib or to the same volume of DMSO (vehicle control) for 1 h. Total cellular lysates were analyzed by Western blotting using the indicated antibodies. Please note that the Western blot B from this Figure and the one from Figure 1B belong to the same experiments and that the Gab2 and β-Actin panels are shown on both Figures.

Using the SILAC-based quantification of the relative abundances of phosphosites (Additional file 2: Table S2 and Additional file 3: Table S3), we next asked which sites were altered in their relative phosphorylation status by more than 2-fold, a common and conservative threshold in phosphoproteomics studies, to discriminate responding from non-responding sites [43], by either IM or DST (Figure 4A-C and Additional file 4: Figure S1 C-E). We observed that IM and DST at equimolar concentrations suppressed the phosphorylation status of 9 sites and 20 sites, respectively. This is also reflected by the drastically reduced recognition of Gab2 by the pan-anti-phosphotyrosine antibody 4G10 (Figure 1A). Interestingly, both inhibitors provoke similar patterns, although in many cases more prominent changes were observed with DST, while Gab2 sites from IM treated cells show only a trend in the same direction (Figure 4A-C and Additional file 2: Table S2 and Additional file 3: Table S3). This observation is in line with the notion that DST is a more potent inhibitor than IM [36] and that phosphorylation of Bcr-Abl at Y177 and CrkL, both widely used as read-outs and biomarkers for Bcr-Abl inhibition [36], are more affected by DST, even at the lower concentration of 0.01 μM (Figure 1A and Figure 5A). Compared to IM, DST significantly affected the phosphorylation status of S2, S132/133, S149, S281, Y491 and S634 of Gab2 at equimolar concentrations (Figure 4C). Interestingly, our previous study in MCF-10A cells revealed that the IM/DST sensitive sites in Gab2 S133, S149 and S281 are also EGF inducible, suggesting that these sites might play a more general role in the regulation of Gab2. In further agreement with our antibody-based analysis in Figure 1, phosphorylation of T391 did not change significantly in the current MS analysis. Taken together, our data show that MS analyses mirror not only phosphorylation data obtained with antibodies, but allow the rapid extension of phospho-maps to the many sites for which antibodies are not available.

**Figure 6:**
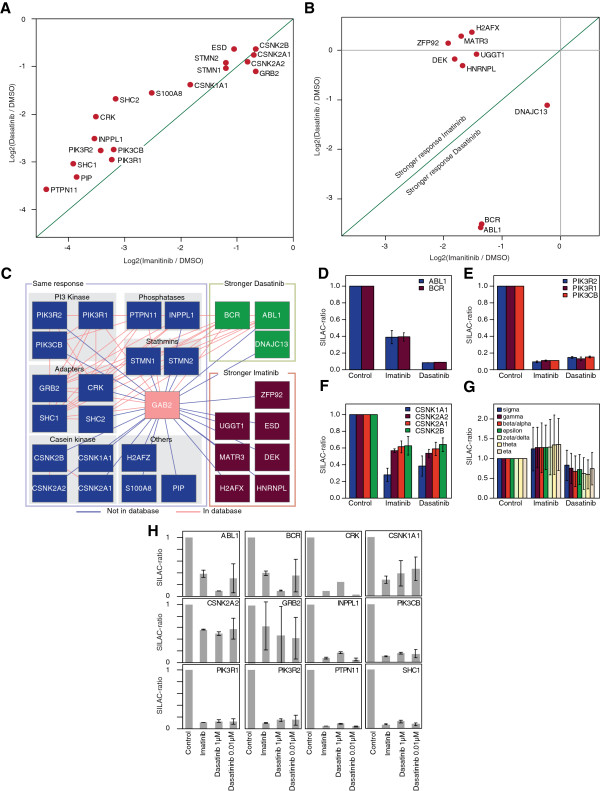
**Imatinib and dasatinib sensitive Gab2 protein interactions.** Scatter plot of proteins showing significant change in the interaction with Gab2 with either (**A**) similar or (**B**) different responses to the imatinib and dasatinib treatments. (**C**) Network diagram of protein-protein interactions of the identified Gab2 interactors. Blue nodes indicate proteins with a similar response to the treatments, green and red nodes are proteins that dissociate stronger in response to Dasatinib or Imatinib, respectively. Red edges indicate protein-protein interactions present in the STRING database (score >0.7) and blue edges indicate putatively novel interactions. Barplot of the observed ratios for (**D**) Bcr-Abl, (**E**) PI3 kinases, (**F**) Casein kinases and (**G**) 14-3-3 protein isoforms, error bars indicate standard deviation of the two biological replicates. (**H**) Significantly depleted proteins highlighted in panels (**A** and **B**) are tested on their sensitivity to different dasatinib concentrations (0.01 and 1 μM).

The stronger impact of DST on the overall phosphorylation status of Gab2 is also reflected by its accelerated electrophoretic mobility compared to the docking protein in lysates from IM or DMSO treated K562 and Bcr-Abl-transformed Ba/F3 cells (Figure 1). However, given the fact that DST targets a broader spectrum of kinases than IM [37,44], including Src-family kinases that drive Gab2 tyrosine phosphorylation in many settings [35,45], it is still surprising that the patterns are quite similar. In full agreement with our previous study [9] and the data in Figure 1, we observe also by MS that IM does not affect one of the three PI3K recruitment sites, pY452, while DST leads to a modest reduction (44%) of this phosphorylation site in K562 cells (Additional file 2: Table S2 and Additional file 3: Table S3). This finding is in line with the observation that both inhibitors do not block Gab2-enhanced AKT phosphorylation in K562 cells (Figure 5A), an event that we previously correlated with TKI resistance in K562 cells and primary CML cells [9]. In contrast to these findings, we also observed a drastic reduction in the interaction of Gab2 to the p85α/β subunits (PIK3R1/2) (Figure 6A, C, E, H and Additional file 5: Table S4), suggesting a stronger influence of IM and DST on the other two PI3K recruitment sites (Y476 and Y584), which we were not able to detect in our MS analysis. For one of the two Shp2 recruitment sites Y643, both drugs reduced the phosphorylation by more than 80% (Additional file 3: Table S3), which could explain their efficacy to block Gab2 enhanced ERK phosphorylation in K562 and Bcr-Abl transformed Ba/F3 cells (Figure 1A, Figure 5A and B).

Finally, we used our SILAC data to compare the spectrum of Gab2 interaction partners in IM or DST treated cells (Additional file 5: Table S4). Protein SILAC ratios were normalized to Gab2 levels, log2 transformed, and ratios of two biological replicates, each, were plotted against each other. Significant outliers in minimally one of the treatments, showing decreased interaction with Gab2 upon treatment, were identified by assuming a normal distribution and calculating Benjamini-Hochberg corrected p values (p < 0.05) using the significance B value of the MaxQuant software [46]. From the identified 1,047 proteins, 18 exhibited similarly reduced interactions in both treatments, 3 reacted stronger in DST treated and 6 in IM treated cells, respectively (Figure 6A and B and Additional file 4: Figure S1A and B). As an internal control, we show that peptides annotated to the Bcr and Abl proteins co-cluster in our graph as they are generated by proteolysis in equimolar proportions from the same Bcr-Abl peptide chain (Figure 6B). We observed a greater loss of Bcr-Abl from Gab2 signalling complexes in cells treated with 1 μM DST compared to cells treated with 0.01 μM DST or IM (Figure 6B-D and H), which might be partially explained by the residual phosphorylation of Bcr-Abl at the Grb2 binding site Y177 in IM compared to DST treated cells. In contrast, the interactions of Gab2 with the seven detected 14-3-3 proteins appear to be largely unresponsive to inhibitor treatments exhibiting no significant alterations (Figure 6G) as also indicated by the stable phosphorylation of S210 and T391 in TKI treated cells (Figure 1).

As shown in Figure 6A and C, IM and DST cause only a modest but significant reduction in the Grb2/Gab2 interaction, which fits well to the observation that this protein-protein interaction is mediated by a SH3 domain and is not supposed to be directly affected by changes in the Gab2 phosphorylation status [15]. However, the fact that the Grb2/Gab2 interaction is still affected by these TKIs could be potentially explained by a recently postulated model in which the tertiary structure of the C-terminal tails of Gab docking proteins is dynamically regulated by phosphorylation events [41,47].

The adaptor proteins SHC1 and SHC2 bind to Gab2 either directly *via* their SH2 domain, or, what has been more consistently observed in several systems, indirectly *via* Grb2 (see [13] for discussion and additional references). However, both potential interaction modes require phosphotyrosine-dependent interactions and therefore the drastic reduction of both SHC proteins in IM and DST treated cells validates our approach (Figure 6A). The role of SHC proteins in Gab signalling complexes remains still unclear, although there is accumulating evidence from other experimental systems that they contribute to the recruitment of the lipid phosphatases SHIP1 and SHIP2 to Gab docking proteins [13]. Indeed, we observe a depletion of SHIP2 (INPPL1) to a similar extent as the SHC proteins in Gab2 signalling complexes purified from IM and DST treated cells (Figure 6A). This finding confirms an earlier study showing that SHIP2 is constitutively phosphorylated and SHC-associated in K562 cells [48].

Furthermore, both TKIs induce a significant reduction of known direct Gab2 interaction partners such as Shp2 (PTPN11) and the p85α/β subunits (PIK3R1/2) as it was also recently described for IM treated K562 cells [38]. Commensurate with the loss of the latter, we noticed a reduction of the catalytic PI3K subunit, p110α, encoded by PIK3CB. Interestingly, p110α and both p85 subunits cluster close to each other (Figure 6A, E, and H), suggesting that they leave the Gab2 signalling complex to a similar extent. We also observe that both TKIs lead to a significant reduction of the Crk adaptor in Gab2 signalling complexes, which is a direct interaction partner of both Bcr-Abl (*via* its SH3 domain) and Gab2 (*via* its SH2 domain; see [13,49] for further details and references). Interestingly, CrkL, which represents the preferred binding partner and substrate of Bcr-Abl [50], was present but not significantly decreased in Gab2 complexes purified from either IM or DST treated cells (Additional file 5: Table S4). This finding suggests that CrkL is predominantly bound by Bcr-Abl in a phosphotyrosine independent manner and might be of interest in the light of CrkL as a commonly used predictive marker for TKI-responsiveness in CML patients [50].

Our analysis also identifies several proteins as significantly decreased by TKI treatment that have not been linked to Gab2 signalling previously (Figure 6A, B, C and F). Although protein abundance differences due to the 4 h of TKI treatment may influence our findings in certain cases, we still regard most interactions as specific due to repeated significant alterations. The first novel TKI-sensitive interaction partners are Stathmin 1 and 2, important regulators of microtubule dynamics. The second represents PIP1, an aspartyl protease. Although PIP1 has been linked by several reports to breast cancer progression, it remains still enigmatic in terms of its precise biological function [51]. Furthermore, its expression in multiple exocrine glandular epithelia, its presence in several bodily fluids and its action on extracellular matrix proteins raises the concern whether the association with Gab2 represents a post-lysis artefact. Nevertheless, this protein has been found to interact with the C-terminus of aquaporin 5 suggesting a potential cytoplasmic localisation [52]. Furthermore, we found PIP1 associated with Gab2 in MCF-10A cells (F.U.W., Adrian Sprenger, J.D and T.B. unpublished data). Future studies will be needed to further validate this interaction. In any case, the detection of PIP1 represents the first report of its expression in CML cells.

We also observe a decrease of the Casein kinase I α (CSNK1A1; CK1) and Casein kinase II α’ (CSNK2A2; CK2) subunits (Figure 6A, C and F), both representing novel interaction partners of Gab2. CK1 and CK2 encode the catalytic subunits of their respective holoenzymes and represent, despite their similar names, members of two different Ser/Thr-kinase subfamilies with distinct consensus phosphorylation sites [53]. Of note, we identified CK2 as a potential kinase being responsible for the phosphorylation of several sites detected in this study (Figure 3). In addition, CK2 is increasingly implicated as a critical oncogenic kinase in several hematopoietic malignancies and solid tumours as well as an activator of the JAK/STAT signalling pathway [54]. Thus, the TKI-sensitive interaction between the Gab2 and CK2 oncoproteins invites for further analyses.

## Conclusions

In summary, our comparative analysis shows that the distinct efficacies of IM and DST are reflected by the differentially altered overall phosphorylation status of the Gab2 protein. Our analysis further underscores that large docking proteins with their extended disordered regions are very rich in phosphorylation sites, whose functions are just starting to emerge [13,41,47,55]. This notion is further supported by recent phospho-proteomic analyses of other large docking proteins such as Gab1 [56,57] or SLP-65/BLNK [58]. Thus, MS-based proteomics represents an ideal method to quantify the complex spatio-temporal phosphorylation patterns of large docking proteins. These detailed phosphorylation catalogues, which are generated by focussing on single protein complexes and are therefore more comprehensive in terms of data depth than global approaches, are also of growing relevance. Indeed, we and others could recently show that disease associated mutations in phosphorylation sites or of critical residues constituting their recognition by cognate kinases are found in various signalling molecules, including several docking protein family members [57,59,60]. Furthermore, MS provides a valuable tool to gain insight into the influence of TKIs on the composition of Gab2 signalling complexes. Given the growing importance of Gab2 in several tumour entities [13,14], we expect that our approach and results will help to shed more light on the various mechanisms by which this docking protein contributes to malignancy.

## Methods

### Transfection and infection of cell lines

Plat-E cells were cultured as described previously [19,61] and transfected using polyethylenimine (Polysciences). Ba/F3 cells were infected with Plat-E culture supernatant as described previously [18]. For the generation of K562/tet cells and their derivatives, 2 × 10^7^ cells were electroporated with 30 μg *Fsp*I-linearized plasmid DNA. Selection of Ba/F3 and K562 cells was commenced 24 h post infection/electroporation (hygromycin B: 0.5 mg/ml, Calbiochem; blasticidin S: 5 μg/ml, Roth; puromycin: 5–7 μg/ml, Sigma).

### Expression vectors

The retroviral pBabe/Bcr-Abl-Hygro vector was derived from pMIG/p210^Bcr-Abl^, a kind gift from Dr. Sebastian Herzog, Albert-Ludwigs-University, Freiburg (Germany). The Bcr-Abl cDNA was excised from pMIG/p210^Bcr-Abl^ using *EcoR*I and subcloned into the *EcoR*I-linearized pBabe-Hygro expression vector. To generate the expression vectors pWZL/Gab2-HA-bsr, Gab2-HA was amplified by PCR from the retroviral pMIG-hGab2-HA [18] and then subcloned into pWZL-Bsr (Addgene). The expression vectors for dox inducible expression of Gab2 in K562tet cells have been described in detail elsewhere [9,32]. Detailed cloning procedures, as well as plasmid sequences, are available upon request.

### Western blotting

K562 and Ba/F3 cells were processed to total cellular lysates by lysing in normal lysis buffer (50 mM TRIS/HCL pH 7.4, 1% Triton-X 100, 137 mM NaCl, 1% Glycerin, 1 mM Natrium Orthovanadate, 0.1 μg/μl Aprotine, 0.01 μg/μl leupeptin, 1mM AEBSF) and analyzed by western blotting as described previously [18].

### SILAC labeling of K562 cells

K562 cells expressing Gab2-HA were cultured in DMEM (Pan Biotech, Aidenbach, Germany) containing 4.5 g/l glucose, Na-Pyruvat, 3.7 g/l NaHCO3, supplemented with penicillin/streptomycin (100 U/ml, 100 μg/ml), glutamine, 10% fetal calf serum (PAA, Coelbe, Germany). Cell populations were labeled for 6 cell doublings with either “light” AA: L-arginine and L-lysine (Arg0, Lys0, Sigma Aldrich), or “medium” AA: L-arginine-^13^C_6_-^14^N_4_ (Arg6; Sigma-Aldrich, Taufkirchen, Germany) and L-lysine-^2^H_4_ (Lys4; Silantes, Munich, Germany), or “heavy” AA: L-arginine-^13^C_6_-^15^N_4_ (Arg10; Sigma-Aldrich) and L-lysine-^13^C_6_-^15^N_2_ (Lys8; Silantes) in SILAC-DMEM media (Thermo Fisher, Langenselbold, Germany), supplemented with L-proline, penicillin/streptomycin (100 U/ml, 100 μg/ml), glutamine, and 10% dialyzed fetal calf serum (Gibco, Paisley, UK) which was used during labeling.

### MS sample preparation

SILAC labelled cells were lysed with normal lysis buffer (50 mM TRIS/HCL pH 7.4, 1% Triton-X 100, 137 mM NaCl, 1% Glycerin, 1 mM Natrium Orthovanadate, 0.1 μg/μl Aprotine, 0.01 μg/μl leupeptin, 1 mM AEBSF), cell nuclei were pelleted and supernatants were used for affinity purification. Gab-HA complexes were enriched using anti-HA-sepharose (Roche Applied Science) in separate IPs, one IP per SILAC label. In the last washing step, sepharose beads were combined and protein complexes eluted by incubating beads for 10 min at 95°C in SDS loading buffer containing 1 mM DTT (Sigma-Aldrich). Reduced samples were alkylated using iodoacetamide (5.5 mM) (Sigma-Aldrich). Protein mixtures were separated by SDS-PAGE (4-12% Bis-Tris gradient gel, NuPAGE (Invitrogen, Karlsruhe, Germany)), gel lanes were cut into 10 slices according to their protein content, samples in-gel digested using trypsin (Promega, Mannheim, Germany), and resulting peptide mixtures were STAGE tipped.

### LC-MS/MS and data processing

Samples for LC-MS/MS were fractionated by nanoscale–HPLC on either an Agilent 1200 or an Eksigent NanoLC-ultra connected online to a LTQ-Orbitrap XL (Thermo Scientific). Peptides were separated over a linear gradient from 10-30% ACN in 0.5% acetic acid with a flow rate of 250 nl/min. All full-scan acquisition was done in the FT-MS part of the mass spectrometers in the range from m/z 350–2000 with an automatic gain control target value of 10^6^ and at resolution 60,000 at m/z 400. MS acquisition was done in data-dependent mode to sequentially perform MS/MS on the five most intense ions in the full scan (Top5) in the LTQ using the following parameters. AGC target value: 5,000. Ion selection thresholds: 1000 counts and a maximum fill time of 100 ms. Wide-band activation was enabled with an activation q = 0.25 applied for 30 ms at a normalized collision energy of 35%. Singly charged and ions with unassigned charge state were excluded from MS/MS. Dynamic exclusion was applied to reject ions from repeated MS/MS selection for 45 s.

All recorded LC-MS/MS raw files (40) were processed together in MaxQuant [46] version 1.3.0.2 with default parameters using the June 2012 UniProt human database which contains 86,898 protein sequences. For databases searching parameters were mass accuracy thresholds of 0.5 (MS/MS) and 20 ppm (precursor), Trypsin/P+DP asprotease, maximum three missed cleavages, carbamidomethylation (C) as fixed modification and oxidation (M), phosphorylation (STY) and protein N-terminal acetylation as variable modifications. MaxQuant was used to filter the identifications for a FDR below 1% for peptides, sites and proteins using forward-decoy searching. Match between runs were enabled with a retention time window of 2 min.

Phosphosites with localization probabilities ≥0.75 (class I sites) were used for bioinformatics analyses.

### Reagents and inhibitors

Imatinib mesylate and dasatinib were purchased from Santa Cruz Biotechnology. Doxycycline hyclate was obtained from Sigma Aldrich. Anti-HA Affinity Matrix was purchased from Roche.

### List of antibodies

Antibodies used in this study were: anti-c-Abl (sc-131), anti-β-Actin (sc-47778) and anti α-Tubulin (sc-23948), all from Santa Cruz, anti-phospho-AKT S473 and T308, anti-p44/p42 MAP kinase, anti-phospho-p44/p42 MAP kinase T202/Y204, anti-phospho-Bcr Y177, anti-Gab2 (26B6), anti-phospho-Gab2 Y452 (C33G1) and S159, anti-CRKL, anti-phospho-CRKL and anti-phospho-STAT5 all from Cell Signaling Technology, anti-HA (3F10) from Roche Molecular Bioscience, anti-phospho-tyrosine (4G10) from Millipore and anti-phospho-Gab2 S210 and T391 antibodies were described previously [19] and obtained from Symansis.

## Abbreviations

AP: Accelerated phase; BC: Blast crisis; CML: Chronic myeloid leukaemia; CP: Chronic phase; Dox: Doxycycline; DST: Dasatinib; HA: Haemagglutinin; IM: Imatinib; MS: Mass spectrometry; SILAC: Stable isotope labelling by amino acids in cell culture; TKI: Tyrosine kinase inhibitor.

## Competing interests

The authors declare that they have no competing interests.

## Authors' contributions

All authors contributed to the design, analysis and discussion of experiments. SH performed all cellular and biochemical experiments. SH, KTGR, BD, CG. and JD conducted the mass spectrometry analyses. TB wrote the manuscript together with SH, KTGR, FUW and JD. TB and JD are co-last authors. All authors reviewed and commented on the manuscript and accepted its final version.

## Supplementary Material

Additional file 1: Table S1 Kinase predictions from NetworKin. Amino acid sequences surrounding phosphorylation sites were employed to predict potential kinases responsible for phosphorylation using the NetworKIN algorithm. Click here for file

Additional file 2: Table S2 Gab2 phosphorylation, imatinib and dastanib compared to DMSO treatment. Phosphosite identification and quantification information is shown. SILAC Gab2 phosphosite ratios of IM and DST treated cells compared to DMSO treated controls are depicted. Click here for file

Additional file 3: Table S3 Gab2 phosphorylation, dastanib compared to imatinib treatment. Phosphosite identification and quantification information is shown. SILAC Gab2 phosphosite ratios of DST treated cells compared to IM treated cells are depicted. Click here for file

Additional file 4: Figure S1 Correlation of protein purification and quantification of biological replicates. **(A-B)** Correlation of protein quantifications of biological replicates for (**A**) imatinib treated cells versus DMSO treated controls and (**B**) dasatinib treated cells versus DMSO treated controls. Significant depleted proteins are annotated in red (sign. B, p < 0.05 BH corrected). **(C-E)** Correlation of phosphosite quantification. Plotted are Gab2 phosphosite ratios normalized to Gab2 protein ratios of (**C**) imatinib versus control, (**D**) dasatinib versus control, and (**E**) dasatinib versus imatinib treated cells of two biological replicates. Click here for file

Additional file 5: Table S5 Gab2 protein-protein interactions, imatinib and dastanib compared to DMSO treatment. Protein identification and quantification information is shown. SILAC ratios of proteins identified in Gab2-HA immuno-precipitations of IM (1 μM) and DST (0.01 μM and 1 μM) treated versus DMSO treated cells are depicted. Proteins exhibiting inhibitor sensitive interactions are highlighted (p<0.05, BH corrected). Click here for file
