# Supplementary figures and images for: Alterations of Gab2 signalling complexes in imatinib and dasatinib treated chronic myeloid leukaemia cells
Source: Cell Commun Signal. 2013 Apr 22;11:30. doi: 10.1186/1478-811X-11-30 (PMC3640961; doi:10.1186/1478-811X-11-30)

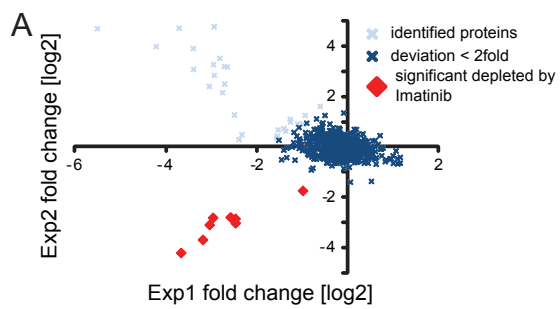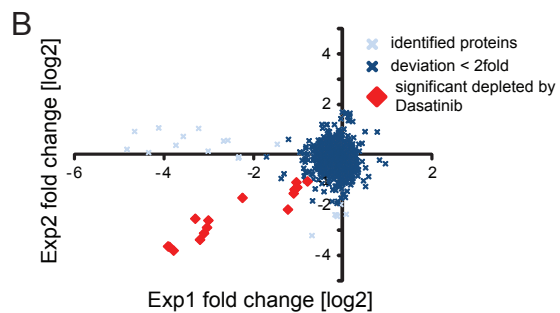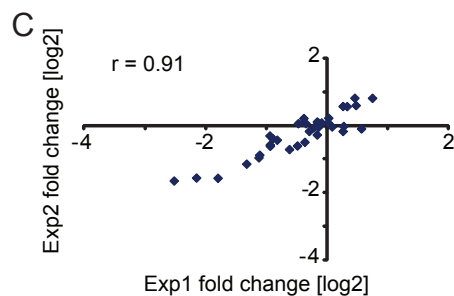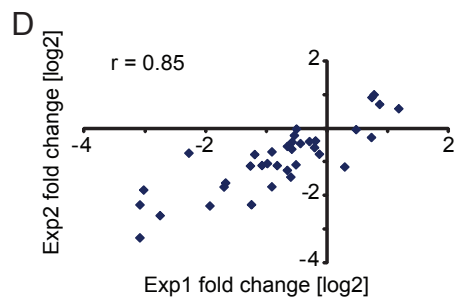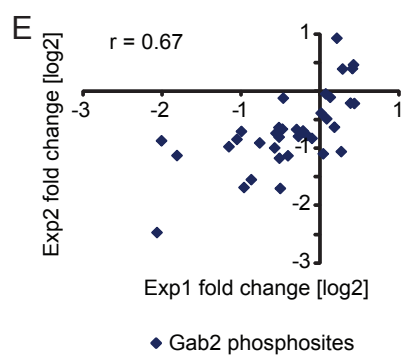

Supplement: Additional file 4: Figure S1 — Correlation of protein purification and quantification of biological replicates. (A-B) Correlation of protein quantifications of biological replicates for (A) imatinib treated cells versus DMSO treated controls and (B) dasatinib treated cells versus DMSO treated controls. Significant depleted proteins are annotated in red (sign. B, p < 0.05 BH corrected). (C-E) Correlation of phosphosite quantification. Plotted are Gab2 phosphosite ratios normalized to Gab2 protein ratios of (C) imatinib versus control, (D) dasatinib versus control, and (E) dasatinib versus imatinib treated cells of two biological replicates. [file 1478-811X-11-30-S4.pdf]
